# Core/shell-type nanorods of Tb^3+^-doped LaPO_4_, modified with amine groups, revealing reduced cytotoxicity

**DOI:** 10.1007/s11051-013-2068-5

**Published:** 2013-10-30

**Authors:** Marcin Runowski, Krystyna Dąbrowska, Tomasz Grzyb, Paulina Miernikiewicz, Stefan Lis

**Affiliations:** 1 Department of Rare Earths, Faculty of Chemistry, Adam Mickiewicz University, Grunwaldzka 6, 60-780 Poznań, Poland; 2 Bacteriophage Laboratory, Institute of Immunology and Experimental Therapy, Polish Academy of Sciences, Rudolfa Weigla 12, 53-114 Wrocław, Poland

**Keywords:** Core/shell nanorods, Cytotoxicity, Surface modification, Amine groups –NH_2_, Luminescence, Tb^3+^*-*doped phosphates

## Abstract

**Abstract:**

A simple co-precipitation reaction between Ln^3+^ cations (Ln = lanthanide) and phosphate ions in the presence of polyethylene glycol (PEG), including post-treatment under hydrothermal conditions, leads to the formation of Tb^3+^-doped LaPO_4_ crystalline nanorods. The nanoparticles obtained can be successfully coated with amorphous and porous silica, forming core/shell-type nanorods. Both products reveal intensive green luminescence under UV lamp irradiation. The surface of the core/shell-type product can also be modified with –NH_2_ groups via silylation procedure, using 3-aminopropyltriethoxysilane as a modifier. Powder X-ray diffraction, transmission electron microscopy, and scanning electron microscopy confirm the desired structure and needle-like shape of the products synthesized. Fourier transform infrared spectroscopy and specific surface area measurements by Brunauer–Emmett–Teller method reveal a successful surface modification with amine groups of the core/shell-type nanoparticles prepared. The nanomaterials synthesized exhibit green luminescence characteristic of Tb^3+^ ions, as solid powders and aqueous colloids, examined by spectrofluorometry. The in vitro cytotoxicity studies reveal different degree toxicity of the products. LaPO_4_:Tb^3+^@SiO_2_@NH_2_ exhibits the smallest toxicity against B16F0 mouse melanoma cancer cells and human skin microvascular endothelial cell lines, in contrast to the most toxic LaPO_4_:Tb^3+^@SiO_2_.

**Graphical Abstract:**

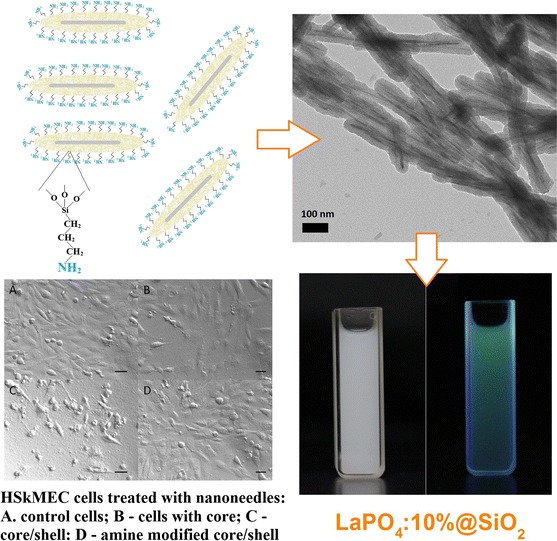

## Introduction

Nanoparticles revealing interesting and useful properties such as luminescence, magnetism, biocompatibility, high-capacity carriers (e.g., drug delivery), resistance for photo-bleaching and thermal-degradation, plasmonic effects, and other optoelectronic properties have been intensively studied over the last decade and their synthesis has been aimed by many researchers (Wang et al. 2010a; Grzyb et al. 2012; Limaye et al. 2011; Yang et al. 2009; Corr et al. 2008; Warren and Chan 2009; Park et al. 2010; Barnes et al. 2003; Zhang et al. 2009). In this group of nanostructures, nanophosphors doped with lanthanide ions exhibiting intensive luminescence, are materials of special importance (Grzyb et al. 2013a). Most of such crystalline, inorganic phosphors are resistant to high temperature and UV irradiation. They also usually do not bleach over a long period of time and they can form stable aqueous colloids (Grzyb et al. 2012), which is very important for bio-applications (Nyk et al. 2008). The properties crucial for bio-applications are the small size, low-toxicity, and intensive luminescence. Lanthanide-doped nanophosphors and Q-dots show such properties. However, both classes of nanoparticles have certain benefits and drawbacks, e.g., some Q-dots based on heavy metals exhibit not only more efficient luminescence than the lanthanide-doped nanomaterials but also higher cytotoxicity (Xu et al. 2011; Gagné et al. 2008). There are many well-known inorganic phosphors based on lanthanide ions, e.g., simple fluorides, borates, vanadates, manganates, phosphates, and their complex analogs. Phosphates with the general formula: LnPO_4_ or hydrated ones (LnPO_4_·*n*H_2_O) doped with activator ions (e.g., Eu^3+^, Tb^3+^, Tm^3+^) can exhibit bright, multicolor luminescence dependent on their crystal structure, chemical composition and morphology (Yu et al. 2006; Phaomei et al. 2010; Phaomei et al. 2011); high refractive index and thermal stability (Ghosh et al. 2010; Blasse and Grabmaier 1994); biocompatibility, which are the well-known properties of many phosphate species (Hirsch et al. 2007). As the nanostructures discussed reveal such properties, the phosphates have been successfully applied fluorescent lamps, tracers, sensors and bio-sensors, contrast agents, drug carriers, and as other biocompatible species (Xu et al. 2011; Hölsä 2009; Kim 2000; Chander 2005; Shionoya and Yen 1999).

Silica is a well-known compound thoroughly studied over last decades (Stöber 1968; Zhao 1998; Jal et al. 2004). Amorphous silica has been commonly applied in chromatography, catalysis, and as a desiccant. Modified nanosilica has been recently used in many bio-applications, e.g., drug delivery, imaging, tracing techniques, and targeting therapies (Slowing et al. 2008; Selvan et al. 2009; Haidar 2010). These applications have been possible because of its inertness, availability, low-cost, ease of fabrication (also on industrial scale), large specific surface area, porosity, simple surface modification, and biocompatibility.

The novel class of nanomaterials, the so called core/shell-type compounds, can combine all the above-mentioned properties of luminescent phase and amorphous silica. When silica is a shell surrounding an internal core, it makes it resistant to oxidation, pH changes, temperature, and many aggressive agents which normally could change the properties of the core or decompose it (Joo et al. 2009; Park et al. 2010; Hu et al. 2010; Runowski et al. 2011). Literature gives many examples of such core/shell-type structures, e.g., nanoparticles with magnetic core like Fe_3_O_4_/SiO_2_ (Correa-Duarte et al. 1998; Stjerndahl et al. 2008), luminescent core NaYF_4_/SiO_2_ (Tan et al. 2010), semiconducting core ZnO/SiO_2_ (Wang et al. 2010b), metallic cores FePt/SiO_2_, Au/SiO_2_ (Warren and Chan 2009; Liz-Marzán et al. 1996), and more complex structures like Fe_3_O_4_/SiO_2_@GdPO_4_:Eu^3+^ or Fe_3_O_4_/SiO_2_/Ag (Runowski et al. 2012; Lv et al. 2010). Thanks to the multifunctional character of these nanomaterials, they can be potentially used in novel, sophisticated systems and techniques in industry, biochemistry, medicine, trace analytics, etc.

In order to make nanomaterials biocompatible and capable of further reactions, usually their surface can be easily modified with functional groups like: –SH, –COOH, –NH_2_, etc. 3-Aminopropyltriethoxysilane (APTES) is a well-known reagent used for introducing amine groups into many compounds. To the best of our knowledge there are two main approaches to the hydrolysis of APTES. The first one demands the use of non-polar, organic solvents like toluene and the absence of water traces in the reaction system (Cauvel and Renard 1997; Mello et al. 2011; Garg et al. 1996). This method is effective but restricted to the use of waterless, cytotoxic organic solvents. The molecules of the solvents can be bound to the silica shell or trapped in its pores, what could be the reason for difficulties with its removal in potential bio-applications. The second approach can be carried out in an ethanol/water system (Deng et al. 2000; Pham et al. 2002; Jung et al. 2012).

Nanomaterials usually exhibit different toxicity than that of the bulk ones or solvated ions, hence their toxicity can be altered, and the crucial factors responsible for the differences are: the size, crystallinity/amorphous, surface charge, ligands present on the surface, etc. (Love et al. 2012; Shaw and Handy 2011). Prior to applications, each nanomaterial should be thoroughly examined against its potential cytotoxicity. Nanosized silica is a representative example of a well-known nanomaterials suitable for numerous of potential applications. The cytotoxicity of silica in micro and submicro size forms is usually negligible, but its nanoforms may often reveal significant cytotoxicity against various cell lines, which has been investigated in vitro and in vivo by many researchers in various biological trials. Previous studies have shown a dose-dependent toxicity of silica nanoparticles. This effect was related to general oxidative stress and apoptosis of nanoparticle-treated cells. Lipid peroxidation and membrane damage were indicated by malondialdehyde and lactate dehydrogenase release. Silica nanoparticles may probably induce genotoxic effects in cells. It is not clear if they act directly by internalization or by cell signaling pathways; however, important changes in the structure of genetic material have been also observed (Lin et al. 2006; Yang et al. 2010; Wang et al. 2007). Surface modification of cytotoxic nanomaterials can be an effective way for altering its toxicity and biocompatibility. As has been originally proposed by Nabeshi et al., nanoparticle surface alternations result in changes in their interactions with surrounding molecules and further, in their biocompatibility with living cells. These changes may influence the processes of internalization and intracellular distribution resulting in a reduction of unfavorable interactions and lower toxicity (Nabeshi et al. 2011).

Here we report a simple approach to the synthesis of a novel, hybrid luminescent nanomaterial composed of small and uniform core/shell-type nanorods, having modified surface with amine groups. The nanostructures obtained reveal bright green luminescence, thanks to the presence of Tb^3+^ ions, and anisotropic morphology. Because of the surface modified with amine groups, they can be easily covalently bound/linked to many organic and biocompatible molecules, via simple organic synthesis reactions. The in vitro cytotoxicity studies have revealed that the level of toxicity of the nanomaterials obtained depends on the type of species present on the surface.

## Experimental

### Materials

Tb_4_O_7_ and La_2_O_3_ (Stanford Materials, 99.99 %) were separately dissolved in a concentrated, nitric acid, HNO_3_ (POCh S.A., ultra-pure) in order to prepare Tb(NO_3_)_3_ and La(NO_3_)_3_ aqueous solutions. Ammonium phosphate monobasic (NH_4_)H_2_PO_4_ (Sigma-Aldrich, ReagentPlus^®^, ≥98.5 %) provided phosphate ions during the synthesis of LnPO_4_. Tetraethyl orthosilicate, TEOS (Sigma-Aldrich, reagent grade, 98 %) was used as a source of silica. 3-Aminopropyltriethoxysilane, APTES (Sigma-Aldrich, ≥98 %) acted as a surface modifier providing aminopropyl moiety. 1-Hexadecyltrimethylammonium bromide, CTAB, and polyethylene glycol 6000, PEG (Alfa Aesar, 98 %) were used as surfactants and structure directing agents. Aqueous solution of 25 % ammonium hydroxide, NH_4_OH (Chempur, pure p.a.) was used for pH adjusting in silanes hydrolysis reactions. In all reactions, ultra-pure distilled water and absolute ethanol were used.

### Synthesis of luminescent nanorods: LaPO_4_:Tb^3+^ 10 %

The synthesis was performed to get 1 g of the product. At first, lanthanide nitrates, Ln(NO_3_)_3_ were mixed at appropriate molar ratio (La^3+^/Tb^3+^ 9/1), and filled with water up to 50 ml. To the prepared solution, 50 ml of absolute ethanol and 0.5 g of PEG 6000 were added. The obtained solution was clear and transparent. The second solution containing 120 % of stoichiometric amount of NH_4_H_2_PO_4_ needed for precipitation of all LnPO_4_ was prepared in the same solvent systems as the previous one, with addition of 0.5 g of PEG 6000 as well. In order to precipitate the doped lanthanide phosphate, the solution containing a source of phosphate was slowly added, drop-by-drop to the first solution. The addition was completed in 30 min. When the precipitation was finished, the whole system was still continuously stirred in ambient conditions, for another 30 min. When the reaction was complete, the precipitate was centrifuged several times and washed with water and ethanol to purify the product. In order to improve the crystallinity of the product, the as-prepared wet precipitate was transferred into Teflon lined vessel and placed in a microwave autoclave (ERTEC, Magnum II, 600 W). The subsequent hydrothermal process lasted for 2 h at 180 °C and 40 bar. After this time, the obtained white, crystalline product was centrifuged several times again and washed with water and ethanol. It is worth noting that after the hydrothermal reaction the wet product irradiated with UV lamp, exhibited intensive green luminescence in contrast to the precipitate before hydrothermal treatment, which did not exhibit any observable luminescence.

### Synthesis of core/shell-type nanorods: LaPO_4_:Tb^3+^ 10 %@SiO_2_

The silica shell was prepared according to the modified Stöber method (Stöber 1968). The as-prepared, hydrothermally treated product—LaPO_4_:Tb^3+^ 10 % (core) was ultrasonicated in water forming a colloidal solution. Afterward, 100 mg of the colloidal core dispersed in a solvent system composed of 40 ml of water, 180 ml of ethanol, and 10 ml of concentrated ammonia (25 %, aqueous solution) were mixed together and ultrasonicated again. Afterward, 200 mg of CTAB was dissolved in the same mixture, in order to improve stability of the colloid formed and acting as a template facilitating the formation of amorphous and porous silica shell. Subsequently, the whole system was ultrasonicated once again. Finally, to the vigorously stirred colloidal solution, 1 ml of TEOS, as a source of silica shell was added. The reaction lasted for 2 h at ambient conditions, upon continuous magnetic stirring. When the reaction was complete the core/shell-type product obtained was left over night in the mother solution. On the next day the product was purified by centrifugation and washed several times with acidic ethanol (95/5 ethanol/HCl) and water. The reason for the use of acidic ethanol was to remove CTAB (template) from silica pores. In order to improve the level of purification, the mixture was ultrasonicated during the washing process. The as-prepared core/shell-type product was dispersed in water forming a colloidal solution.

### Surface modification: LaPO_4_:Tb^3+^ 10 %@SiO_2_@NH_2_

A portion of 25 mg of colloidal LaPO_4_:Tb^3+^ 10 %@SiO_2_ was dispersed in 2 ml of water with the use of ultrasounds. Afterward 45 ml of ethanol and 5 ml of 25 % aqueous ammonia solution were added to the colloid prepared. The system was vigorously stirred at ambient conditions. Subsequently, 0.1 ml of APTES and 0.1 ml of TEOS were injected into the prepared colloid. The reaction was continued for 3 h, at 50–60 °C. When the reaction was complete, the products were purified by centrifugation and washed several times with water and ethanol. Part of the final product was dispersed in water forming a colloidal solution, and the rest of the product was dried in vacuum for measurements.

**Fig. 1:**
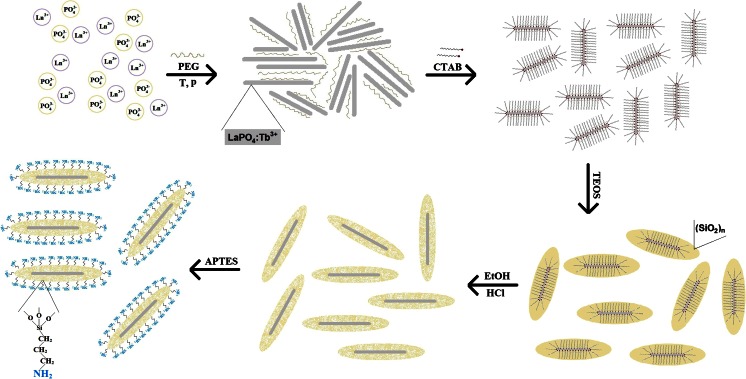
Scheme of formation of LaPO_4_:Tb^3+^ 10 %@SiO_2_@NH_2_—surface-modified core/shell-type nanorods

Figure 1 shows a scheme of the formation of nanorods, their coating with silica and subsequent surface modification with amine groups.

## Characterization

Electron microscopy measurements were performed using a Transmission Electron Microscope LEO 912AB, ZEISS operating at accelerating voltage 120 kV, and a Scanning Electron Microscope LEO 435VP, ZEISS operating at 3 kV. The nitrogen adsorption–desorption isotherms recorded at 77 K were measured using a Quantachrome NOVA 1000e apparatus. Specific surface area, pore volume and pore size distribution were calculated by the Brunauer–Emmett–Teller (BET) and Barrett–Joyner–Halenda (BJH) methods, respectively. Before measurements, all samples were preheated at 300 °C overnight in vacuum. Powder X-ray diffractograms (XRD) were recorded on a Bruker AXS D8 Advance diffractometer, using CuKα radiation (*λ* = 1.5406 Å), accelerating voltage 40 kV and emission current 40 mA. IR spectra were measured with an Fourier transform infrared (FT-IR) spectrophotometer, JASCO 4200. The IR spectra were measured in the transmission mode, the samples were mixed with KBr, ground, and pressed into pellets. The excitation and emission spectra including luminescence decay curves (dried, solid powders) were carried out with a Hitachi F-7000 spectrofluorometer in ambient conditions. The excitation and emission spectra were appropriately corrected for the apparatus response.

### The cytotoxicity of nanorods

Cytotoxicity of the nanoparticles investigated was tested in proliferation assays and by cell imaging. Both cancer cells and normal cells were used. The human skin microvascular endothelial (HSkMEC) cell line was obtained from Cell Culture Collection of the IIET (Wroclaw, Poland). The B16F0 mouse melanoma cancer cell line was obtained from the American Type Culture Collection (ATCC, USA). Both lines are kept at the Cell Culture Collection of the IIET (Wroclaw, Poland). The cells were cultured in OptiMEM (Invitrogen, Cergy Pontoise, France) supplemented with 5 % fetal bovine serum, 40 μg/ml gentamycin (Invitrogen) and 0.05 μg/ml fungizone (Invitrogen). The cells were seeded at 5 × 10^4^ cells/cm^2^ at 96-well plate or 6-well plate 2 h before experiments and maintained at 37° in a 5 % CO_2_/95 % air atmosphere during the incubation prior to the experiment. Final concentrations of the nanoparticles were: 0.005, 0.01, 0.05, 0.1, 0.5, 1 mg/ml. All cultures including controls were supplemented with PBS to the same final volume. The effect of nanorods on the cells was assessed as follows: (i) cell condition and morphology was assessed by optical microscopy in sequential imaging, (ii) total cell material production was assessed by SRB assay (sulforhodamine B assay). The details of this technique were described by Skehan et al. (1990). The assay was performed after 48-h exposure of the cultured cells to the agents tested. The cells attached to the plastic were fixed by gently layering cold 50 % TCA (trichloroacetic acid, Aldrich-Chemie, Germany) on the top of the culture medium in each well. The plates were incubated at 4 °C for 1 h and then washed five times with tap water. The background optical density was measured in the wells filled with culture medium, without the cells. The cellular material fixed with TCA was stained with 0.4 % sulforhodamine B (SRB, Sigma, Germany) dissolved in 1 % acetic acid (POCh, Gliwice, Poland) for 30 min. Unbound dye was removed by rinsing with 1 % acetic acid. The protein-bound dye was extracted with 100 μl 10 mM unbuffered tris base (POCh, Gliwice, Poland) for determination of optical density (at 540 nm) in a computer-interfaced, 96-well microtiter plate reader Multiskan RC photometer (Labsystems, Helsinki, Finland). Each nanoparticle type and concentration in each cell line culture was tested two or three times, each test in 12-well groups, one exemplary experiment results of each type was presented (*N* = 12). The results were analyzed by ANOVA with the Statistica 8.0 software package (www.statsoft.pl).

**Fig. 2:**
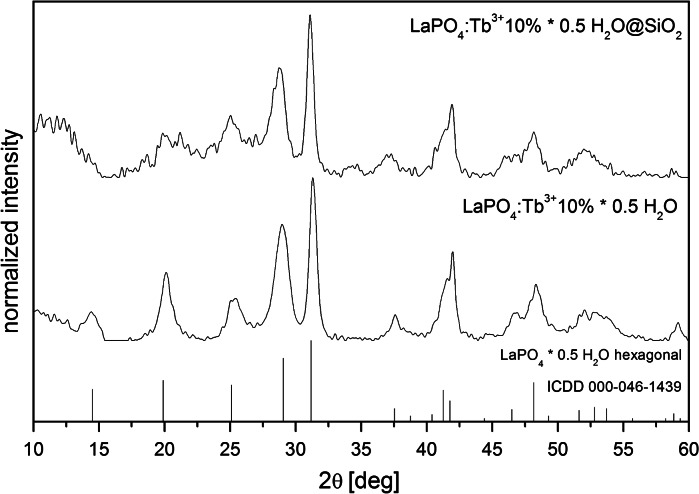
XRD patterns of LaPO_4_:Tb^3+^ 10 % (·0.5H_2_O) and LaPO_4_:Tb^3+^ 10 % (·0.5H_2_O)@SiO_2_ nanostructures

## Results and discussion

### Structure and morphology

The experimental X-ray powder diffractograms of LaPO_4_:Tb^3+^ 10 % and LaPO_4_:Tb^3+^ 10 %@SiO_2_ were compared with pattern from International Centre for Diffraction Data (ICDD) standards database (Fig. 2). Both diffractograms are similar and fit well to the XRD pattern, number 000-046-1439, corresponding to the hexagonal, hydrated lanthanum phosphate, LaPO_4_·0.5H_2_O (for the reader convenience, compounds hydration is neglected in all formulas within the article). The diffractogram of core/shell-type product reveals an extra broad reflex in the 2*θ* range of 10°–15°, characteristic of amorphous silica (Musić et al. 2011). Both obtained powder XRD have broadened reflexes, which indicates the nanocrystallinity of the products.

Table 1 presents a comparison of cell parameters from reference database (hydrated LaPO_4_) with the results of calculations based on XRD of the product obtained (doped LaPO_4_:Tb^3+^ 10 %). As follows from comparison of the data, a decrease in the unit cell volume takes place after Tb^3+^ doping. The reason for this phenomenon is the ionic radius of Tb^3+^ (dopant) smaller than that of La^3+^ ion (host). The above observation also confirms successful substitution of Ln^3+^ ions in the crystalline product.

**Table 1:** Comparison of crystal lattice parameters from reference database (ICDD 000-046-1439) and XRD data of synthesized LaPO_4_:Tb^3+^ 10 % nanorods

Data	*a* (Å^3^)	*c* (Å^3^)	Cell volume (Å^3^)
Reference	7.100	6.494	283.5
XRD	7.104 (6)	6.479 (7)	283.2 (4)

**Fig. 3:**
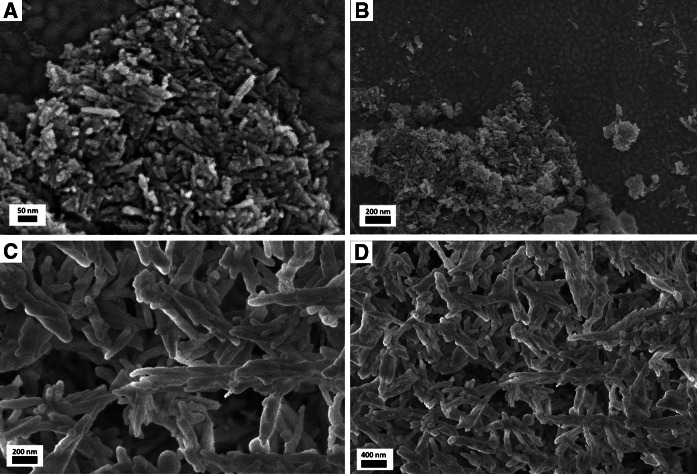
SEM images of LaPO_4_:Tb^3+^ 10 %—core (**a**, **b**) and LaPO_4_:Tb^3+^ 10 %@SiO_2_—core/shell (**c**, **d**) nanorods

The scanning electron microscopy (SEM) images presented in Fig. 3 reveal a uniform, needle-like shape of all nanoparticles of the core (a, b) and core/shell-type (c, d) products. The clusters of nanoparticles are in the range of nanometers. However, the core/shell-type products consist of larger nanostructures, because of the presence of an external silica shell. It is also worth mentioning that there is no any other phase present among the core/shell-type product’s nanoparticles. In other words, the coating of lanthanum phosphate nanorods with silica is successful and uniform, without the presence of spherical particles in the final nanomaterial, characteristic of pure silica.

**Fig. 4:**
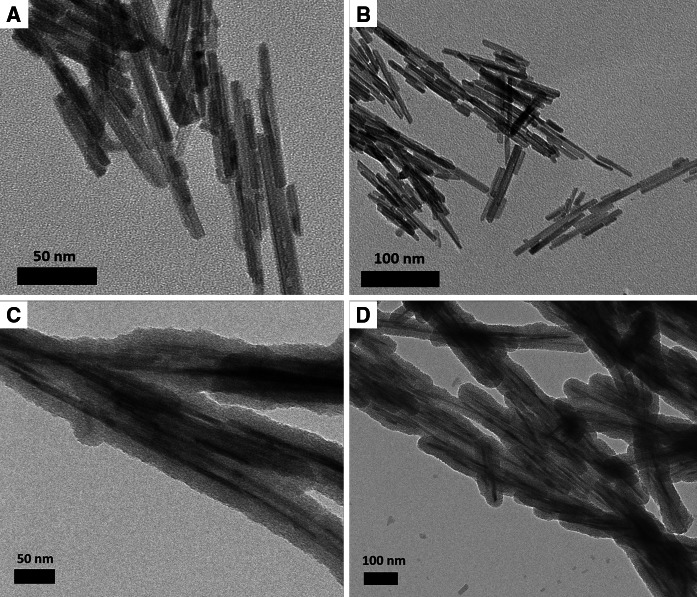
TEM images of LaPO_4_:Tb^3+^ 10 %—core (**a**, **b**) and LaPO_4_:Tb^3+^ 10 %@SiO_2_—core/shell (**c**, **d**) nanorods

The next set of pictures (Fig. 4) shows transmission electron microscopy (TEM) images of the core (a, b) and core/shell-type (c, d) products. The average dimensions of LaPO_4_:Tb^3+^ 10 % are the width in the range of 7–11 nm and length of 80–110 nm. The bottom images (c, d) show a clear evidence of formation of a thin (15–20 nm) external silica shell, deposited on crystalline cores. What is also important, there are no traces of bare silica spheres among the core/shell-type nanorods. It means that the synthesized nanomaterial is homogeneous and uniform, which is important in many applications.

**Fig. 5:**
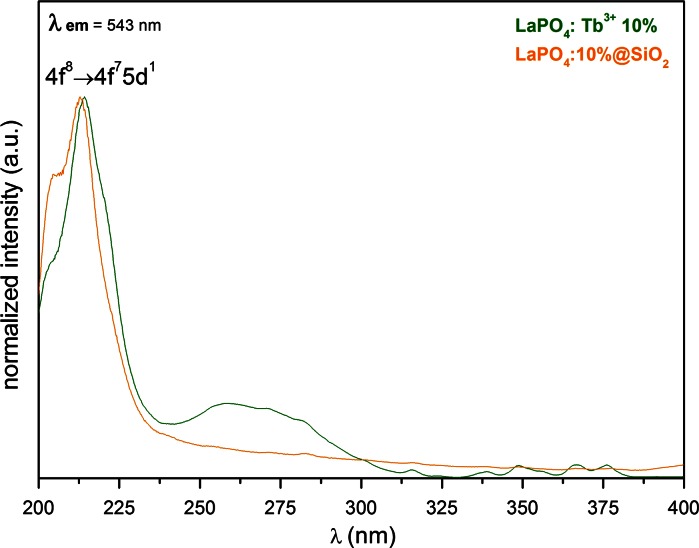
Excitation spectra of LaPO_4_:Tb^3+^ 10 % and LaPO_4_:Tb^3+^ 10 %@SiO_2_, recorded for dried samples

**Fig. 6:**
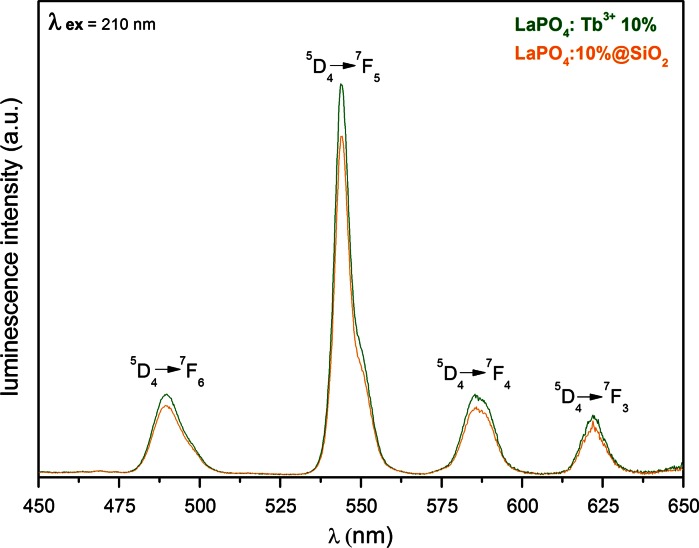
Emission spectra of LaPO_4_:Tb^3+^ 10 % and LaPO_4_:Tb^3+^ 10 %@SiO_2_, recorded for aqueous colloids

**Fig. 7:**
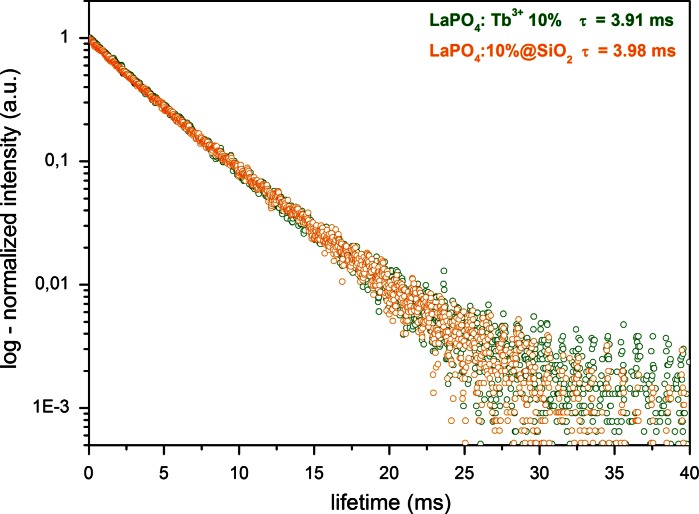
Luminescence decay curves of LaPO_4_:Tb^3+^ 10 % and LaPO_4_:Tb^3+^ 10 %@SiO_2_ observed at *λ*_em_ = 543 nm and *λ*_ex_ = 210 nm

Figures 5, 6, and 7 illustrate the spectroscopic properties of the core and core/shell-type products. The spectroscopic studies (emission spectra and luminescence lifetimes) of the nanostructures obtained were performed in colloidal state (aqueous colloids), preserving the same amount of the luminescent phase in a fixed volume of a given colloid, in order to compare its relative emission intensity. Such measurements, allow the studies of luminescence quenching effect by water molecules. The dried products (powders) were used for the measurements of excitation spectra, because the spectra of the colloids were difficult for interpretation. Figure 5 presents the excitation spectra of the compounds studied recorded at *λ*_em_ = 543 nm (the center of Tb^3+^ emission main peak). Both spectra reveal the same dominant band in the range of 210–214 nm, corresponding to the allowed 4f^8^ → 4f^7^5d^1^ transition within Tb^3+^ ions. The slight change in its position and shape in the spectra of core/shell products is due to the absorption of silica present in the system. The minor peaks in the higher wavelengths correspond to the forbidden f → f transitions in Tb^3+^ ions. Again, because of the silica absorption in this region, these peaks are not observable in the core/shell spectrum.

Figure 6 presents the emission spectra of the core and core/shell nanophosphors (*λ*_ex_ = 210 nm), revealing four narrow bands, typical of Tb^3+^-doped phosphors, namely: ^5^D_4_ → ^7^F_6_, ^5^D_4_ → ^7^F_5_, ^5^D_4_ → ^7^F_4_, ^5^D_4_ → ^7^F_3_, related to magnetic dipole transitions within 4f shell of Tb^3+^ ions (Lis 2002). Both spectra exhibit the same shape because of magnetic dipole nature of the transitions in Tb^3+^ ions, which are not sensitive to the site symmetry of the emitting ion. Their relative intensities are similar, however, the total intensity of luminescence of the bare core—LaPO_4_:Tb^3+^ 10 % is slightly higher. We assume that this decrease is related to the thin silica shell on the surface of emitting nanorods which scatter and absorb light (Grzyb et al. 2013b).

Figure 7 shows the luminescence decay curves and calculated radiative lifetimes for the ^5^D_4_ → ^7^F_5_ transition of the Tb^3+^ ions. The data were collected at 293 K, *λ*_em_ = 543 nm and *λ*_ex_ = 210 nm, for both products. The profiles recorded are fitted by a monoexponential decay, (*τ*), *y* = *A*1 × exp(−*x*/*t*) + *y*0. In hexagonal lanthanum phosphate, all lanthanide ions are situated in the same type of coordination site, which is confirmed by monoexponential decay of the luminescence. The calculated values of lifetimes are 3.91 and 3.98 ms for the core and core/shell-type products, respectively. These values are in good correspondence to the relatively long lifetimes of lanthanide-doped inorganic phosphors (Omkaram et al. 2008; Grzyb et al. 2012). An explanation of the slight increase in lifetime of the core/shell-type product can be a protective effect of the external thin layer of the silica shell, which decreases the quenching of the excited states of Tb^3+^ ions by O–H oscillators of water molecules (Wu et al. 2011).

**Fig. 8:**
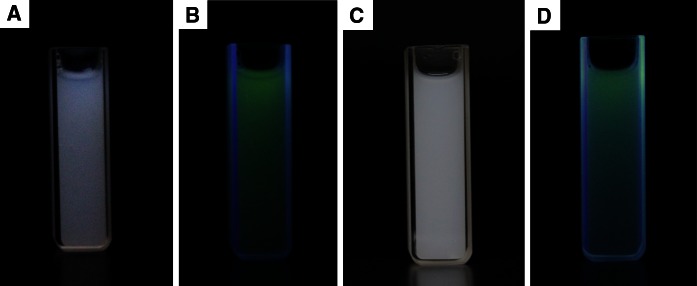
Photographs of the aqueous colloids of LaPO_4_:Tb^3+^ 10 %—core (**a**, **b**) and LaPO_4_:Tb^3+^ 10 %@SiO_2_—core/shell (**c**, **d**), taken under ambient light (**a**, **c**) and under 254 nm UV light (**b**, **d**)

Figure 8 presents the previously discussed aqueous colloids of LaPO_4_:Tb^3+^ 10 % and LaPO_4_:Tb^3+^ 10 %@SiO_2_ are presented. They were prepared by ultrasonication of the products in water, and as mentioned before, the same concentration/amount of the luminescent phase was used for both dispersions. Colloidal solutions of both nanostructures are stable, and the colloidal particles do not settle down for a few days. Photographs on the left side present colloids of the core (a, b) and those on the right side present colloids of the core/shell-type product (c, d), taken under day light (a, c) and under UV light, 254 nm (b, d). Both of them exhibit bright, green luminescence characteristic of Tb^3+^ ions. In the core/shell-type compound, the silica shell scatters light and that is why its emission reveals a bit different hue.

### Surface modification

In order to show that a modification of the surface of core/shell-type, LaPO_4_:Tb^3+^ 10 %@SiO_2_ nanoparticles is feasible and simple to perform, the surface was modified with –NH_2_ groups. The reasons for such a choice were: multifunctional character of amines, i.e., biocompatibility, the use for water purification from heavy metals, the use as chelating agents, as reagents in many chemical reactions, a possibility of linking/binding to other organic molecules, availability of literature data on modifications with –NH_2_ groups, a possibility of further modifications based on organic synthesis reactions. The reaction was carried out in a polar, non-toxic ethanol/water solvent system. However, the hydrolysis of APTES in a system without TEOS was ineffective as evidenced by the absence of the absorption peaks assigned to –NH_2_ and –CH_2_ groups in the IR spectra (data not shown). This fact is also described in literature (Bagwe et al. 2006), which is in agreement with the results of our study, therefore we used a mixture of APTES and TEOS for the surface modification. Their hydrolysis and subsequent co-condensation permitted successful modification of the core/shell nanomaterial surface with amine groups (≡Si–(CH_2_)_3_–NH_2_).

The crystalline structure, shape and spectroscopic properties of the core/shell-type nanoparticles obtained, were not changed after surface modification with amine groups. The presence of amine groups was checked by FT-IR spectroscopy, analysis of specific surface area of the products and their cytotoxicity.

**Fig. 9:**
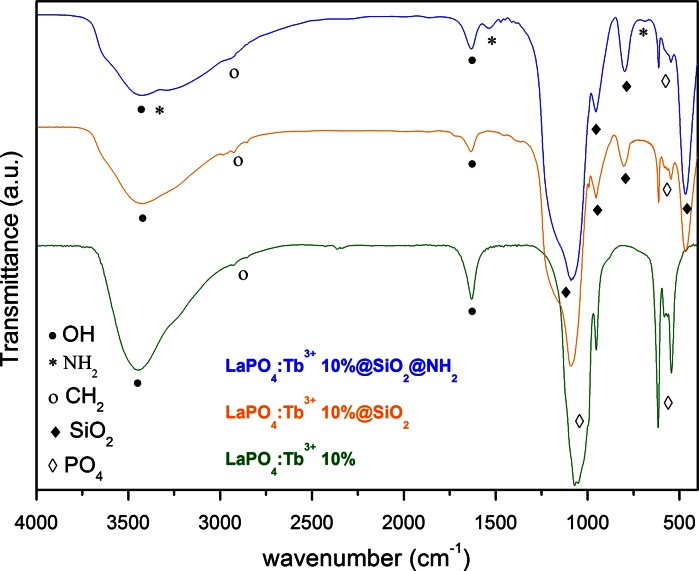
FT-IR spectra of LaPO_4_:Tb^3+^ 10 %, LaPO_4_:Tb^3+^ 10 %@SiO_2_, and amine-modified LaPO_4_:Tb^3+^ 10 %@SiO_2_@NH_2_ nanomaterials

Figure 9 presents the IR spectra of LaPO_4_:Tb^3+^ 10 %, LaPO_4_:Tb^3+^ 10 %@SiO_2_ and LaPO_4_:Tb^3+^ 10 %@SiO_2_@NH_2_. All spectra reveal broad absorption peaks centered at 3,440 and 1,640 cm^−1^ corresponding to the O–H stretching (*ν*) and deformation (*σ*) vibrations, respectively. The O–H bonds come from water adsorbed on the surface and inside the mesopores of silica as well as silanol groups of amorphous silica. Absorption peaks around 2965, 2924, and 2855 cm^−1^ are assigned to *ν*C–H vibrations of –CH_2_ groups of PEG molecules (adsorbed on the surface of phosphate nanorods) and aminopropyl moiety (after APTES modification). All the spectra show also very intensive peaks corresponding to the vibrations of phosphate groups (partially overlapping with bands from silica) at 1049, 951 and at 615, 542 cm^−1^ and they are assigned to the stretching and bending vibrations within PO_4_ groups, respectively (Xu et al. 2011; Lucas et al. 2004). In the spectra of core/shell-type product and APTES-modified one, three bands related to the silica, are visible, and they are assigned to the following vibrations: *ν*Si–O–Si(asym) at 1178,1085 cm^−1^, *ν*Si–O– at 952 cm^−1^, *ν*Si–O–Si(sym) at 800 cm^−1^, and *σ*O–Si–O at 467 cm^−1^ (Musić et al. 2011; Mello et al. 2011; Wang et al. 2010c). In the spectrum of the amine-modified product, the following additional peaks are observed: around 3,400 and 3,300 cm^−1^ assigned to *ν*N–H vibrations (overlapping with *ν*O–H vibrations), at 1,540 cm^−1^ *σ*N–H (scissoring) and at 690 cm^−1^ *σ*N–H (wagging) (Mello et al. 2011; Wang et al. 2010c). The presented spectra undoubtedly confirm the presence of silica shell and its successful modification with amine groups.

**Fig. 10:**
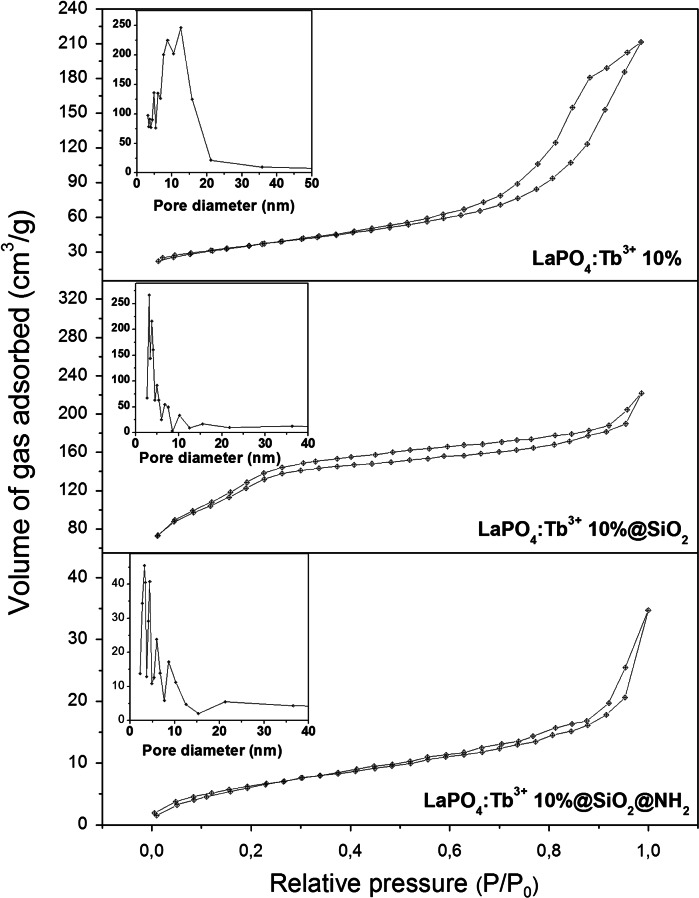
Nitrogen adsorption–desorption isotherms and pore size distribution insets of LaPO_4_:Tb^3+^ 10 %—core, LaPO_4_:Tb^3+^ 10 %@SiO_2_—core/shell, and amine-modified LaPO_4_:Tb^3+^ 10 %@SiO_2_@NH_2_ hybrid, core/shell-type nanostructures

Specific surface area and porosity of the products were examined by nitrogen adsorption–desorption method. Figure 10 presents the N_2_ adsorption–desorption isotherms recorded for the nanostructures studied in the temperature range 77–573 K. According to BET method, the specific surface area of LaPO_4_:Tb^3+^ 10 % nanorods is 126.100 m^2^/g, while that of the core/shell-type product—LaPO_4_:Tb^3+^ 10 %@SiO_2_ it is equal to 431.725 m^2^/g, and that of LaPO_4_:Tb^3+^ 10 %@SiO_2_@NH_2_ it is 34.518 m^2^/g. All compounds reveal N_2_ sorption isotherms characteristic of mesoporous materials, namely type IV isotherm for both core/shell-type products, and type V isotherm for core nanomaterial (Sing et al. 1985). These values are in a good agreement with predictions, because they reflect an increased surface area of the core/shell-type product after its coating with porous silica which has high surface area (usually hundreds of m^2^/g). Inorganic compounds have usually small specific surface area, in the range from several to several tens of m^2^/g. In contrast, LaPO_4_:Tb^3+^ 10 % nanorods reveal prominent surface areas, which reflects small size of its nanocrystals, large surface area to volume ratio and porosity. On the other hand, a significantly decreased specific surface area of the LaPO_4_:Tb^3+^ 10 %@SiO_2_@NH_2_, confirms a successful modification of its surface and the presence of pores filled with the silanes applied. The total pore volume is similar for the core and core/shell-type nanomaterials, namely 0.327 and 0.343 cm^3^/g for the core and core/shell-type product, respectively. These values are typical of inorganic, porous compounds. However, for the core/shell modified with –NH_2_ groups, the total pore volume is much smaller and equals to 0.054 cm^3^/g. This decrease in the pore volume is probably related to the presence of covalently bound amine groups inside the pores and on the surface of the core/shell-type nanorods (linked by propyl moiety). The distribution of pore size (Fig. 10 insets) is calculated by BJH method. The average pore diameter in LaPO_4_:Tb^3+^ 10 % is 10.371 nm, for the core/shell-type product it is equal to 3.179 nm, and for the amine-modified core/shell-type nanomaterial it is 6.766 nm. Crystalline nanorods of the core are featured with mesopores, which can be attributed to the interstitial spaces/gaps between nanorods (Xu et al. 2011). Both, the core/shell-type product and the amine-modified one, have smaller pores, which is typical of compounds based on porous silica. The average pore diameter for the amine-modified nanomaterial is bigger than for the unmodified core/shell, because of a broader distribution of its pore diameter. We assume that this phenomenon is related to the formation of some additional superficial pores as a result of surface modification.

In order to determine the amount of amine groups in the obtained nanomaterials the elemental analysis of LaPO_4_:Tb^3+^ 10 %@SiO_2_, and LaPO_4_:Tb^3+^ 10 %@SiO_2_@NH_2_ was performed. For the LaPO_4_:Tb^3+^ 10 %@SiO_2_ product the obtained content of N, C, H was 0.021, 3.449, and 2.417 (wt%), respectively, whereas for amine-modified product, LaPO_4_:Tb^3+^ 10 %@SiO_2_@NH_2_ the obtained content of N, C, H was 2.902, 7.839, and 3.373 (wt%), respectively. On the basis of the results, the molar concentration of amine groups was determined for 2.058 mmol per one gram of the product. The results additionally confirmed the successful surface modification of the synthesized LaPO_4_:Tb^3+^ 10 %@SiO_2_@NH_2_ nanomaterial and large amount of amine groups present in its structure.

### The effect of nanorods on mammalian cells in vitro

The effect of nanorods on mammalian cells (B16F0 and HSkMEC) was investigated by cell proliferation studies in in vitro cultures. In these conditions, the cells were directly exposed to interactions with nanorods, without physiological co-factors or background. 48-h exposition of the cells to nanorods revealed substantial differences in the cell sensitivity to the three products investigated: LaPO_4_:Tb^3+^ 10 %, LaPO_4_:Tb^3+^ 10 %@SiO_2_, and LaPO_4_:Tb^3+^ 10 %@SiO_2_@NH_2_. In general, mammalian cells were most vulnerable to the core/shell-type product; however, surface modification with NH_2_ groups resulted in an important increase in safety and substantial decrease in attenuating effect on the cells.

**Fig. 11:**
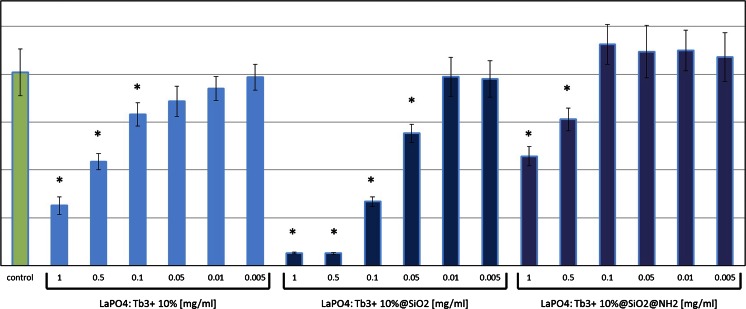
Cell viability in the cultures treated with nanorods evaluated by SRB assay on B16F0 cells. *Bars* present 540 nm absorbance in the assay reflecting the amount of cell material in the cultures treated with gradient concentrations of LaPO_4_:Tb^3+^ 10 % or LaPO_4_:Tb^3+^ 10 %@SiO_2_ or LaPO_4_:Tb^3+^ 10 %@SiO_2_@NH_2_. Control cell cultures were supplemented with adequate volume of PBS. Groups of wells that significantly differ from the control wells (by ANOVA) are marked with *asterisks*

**Fig. 12:**
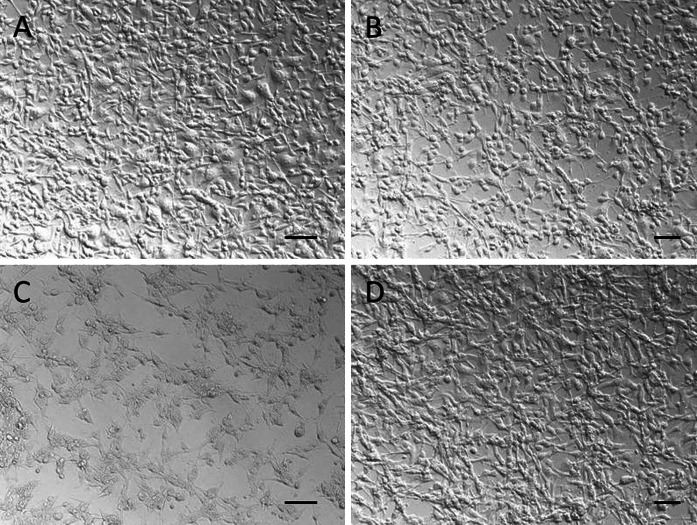
Microscopic images of 48-h culture of B16F0 cells treated with nanorods: **a** control cells; **b** cells cultured with 0.05 mg/ml LaPO_4_:Tb^3+^ 10 %; **c** cells cultured with 0.05 mg/ml LaPO_4_:Tb^3+^ 10 %@SiO_2_; **d** cells cultured with 0.05 mg/ml LaPO_4_:Tb^3+^ 10 %@SiO_2_@NH_2_ (*scale bars* represent 500 μm)

**Fig. 13:**
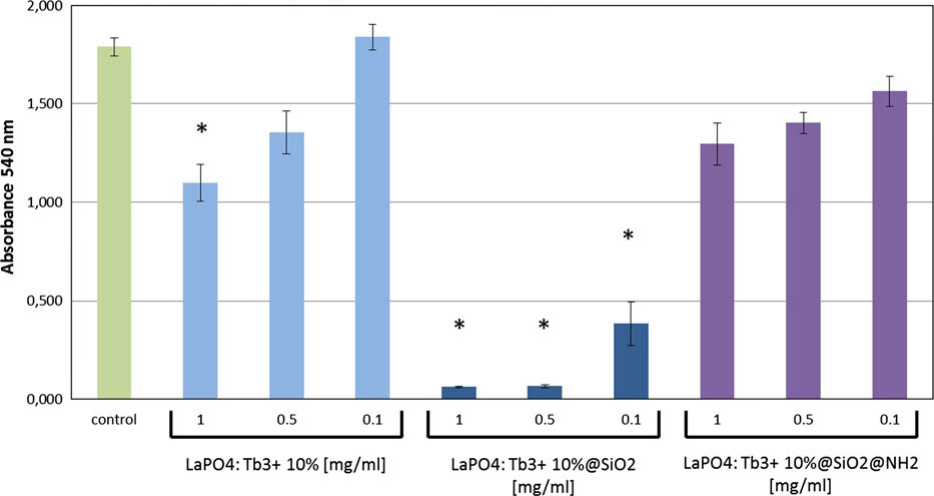
Cell viability in the cell cultures treated with nanorods studied by SRB assay on HSkMEC cells. *Bars* present 540 nm absorbance in the assay reflecting the amount of cell material in the cultures treated with gradient concentrations of LaPO_4_:Tb^3+^ 10 % or LaPO_4_:Tb^3+^ 10 %@SiO_2_ or LaPO_4_:Tb^3+^ 10 %@SiO_2_@NH_2_. Control cell cultures were supplemented with adequate volume of PBS. Groups of wells that significantly differ from the control wells (by ANOVA) were marked with *asterisks*

**Fig. 14:**
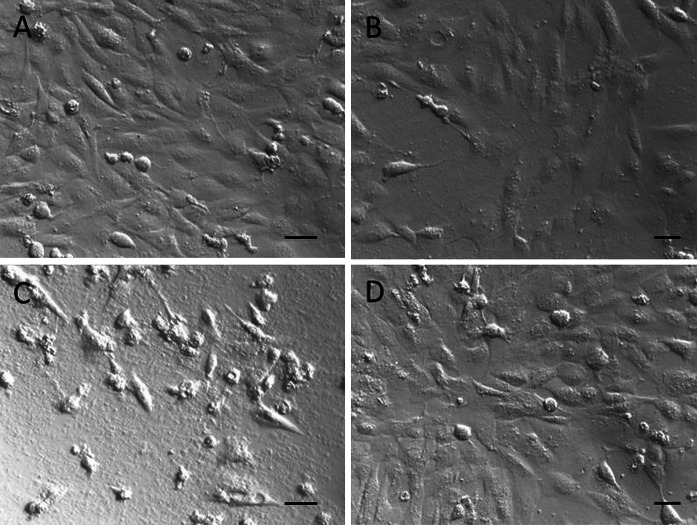
Microscopic images of 48-h culture of HSkMEC cells treated with nanorods: **a** control cells; **b** cells cultured with 0.05 mg/ml LaPO_4_:Tb^3+^ 10 %; **c** cells cultured with 0.05 mg/ml LaPO_4_:Tb^3+^ 10 %@SiO_2_; **d** cells cultured with 0.05 mg/ml LaPO_4_:Tb^3+^ 10 %@SiO_2_@NH_2_ (*scale bars* represent 250 μm)

Potential toxic effects of LaPO_4_:Tb^3+^ 10 %, LaPO_4_:Tb^3+^ 10 %@SiO_2_, and LaPO_4_:Tb^3+^ 10 %@SiO_2_@NH_2_ were assessed by determination of their tolerable concentrations for B16F0 cultures. These concentrations were: 0.05 mg/ml of non-modified LaPO_4_:Tb^3+^ 10 %, 0.01 mg/ml of the core/shell-type product, while as much as 0.1 mg/ml of the core/shell modified with NH_2_ groups was (Fig. 11). Further, microscopic imaging of the cultures revealed dramatic changes in B16F0 cell number and morphology in the group treated with unmodified core/shell products already in concentrations 0.05 mg/ml. In the same conditions only minor changes were noticed in the group treated with 0.05 mg/ml of LaPO_4_:Tb^3+^ 10 % or LaPO_4_:Tb^3+^ 10 %@SiO_2_@NH_2_ (Fig. 12). Visualization of the cell condition suggests that the exposure of cells to unmodified core/shell-type products leads not only to the loss of cell viability and their slower growth, but rather to substantial membrane damage, which results in an apoptotic cell death. Similar effects were observed in HSkMEC cell cultures, which were most sensitive to toxic activity of silica shell products, but they tolerated well the same products modified with amine groups (Figs. 13, 14).

These observations are in line with a previous report of Nabeshi et al. (2011) who investigated model spherical silica nanoparticles and presented toxic effects of external silica shell, which were decreased by surface modification with amine groups. He used one cell line of murine macrophages, the range of silica products was 0.01–1 mg/ml. In our studies, the unmodified silica product was also the most toxic in two different cell lines. The remained residues of CTAB in the structure of silica could potentially contribute to increasing toxicity of LaPO_4_:Tb^3+^ 10 %@SiO_2_ (Zhang et al. 2012). However, we assume that the mentioned effect can be negligible or of minor influence, because of the large specific surface area of the mentioned product, which confirms that the template agent used has been successfully removed (CTAB was removed by washing the product several times with acidic ethanol, assisted with ultrasounds) from the silica pores located near the surface of the discussed core/shell-type nanomaterial. The surface modification with amine groups resulted in a product of approximately 10-times lower toxicity in comparison to the non-modified LaPO_4_:Tb^3+^ 10 %@SiO_2_. The amine coated surface was also better tolerated by the cells than the “bare” LaPO_4_:Tb^3+^ 10 % (core). Surface alternations were postulated to change biological nature of small silica nanoparticles interactions with surrounding molecules, e.g., serum proteins or culture medium compounds. The surface modification may influence the processes of internalization and intracellular distribution resulting in a decrease in the unfavorable interactions and lower toxicity (Nabeshi et al. 2011). Our results show that the core/shell-type nanorods with the amine-modified silica shell can also be a better choice for biological applications. They are good candidates for further modifications, e.g., with targeting peptides or other biologically active organic compounds, but they are also the safer form of the products to which the mammalian cells show the highest tolerability.

## Conclusions

Tb^3+^-doped crystalline nanorods, exhibiting green luminescence, have been synthesized under hydrothermal conditions. Subsequently their surface has been coated with silica, forming the core/shell-type nanorods, which were further successfully modified with amine groups. The in vitro cytotoxicity studies unambiguously revealed significant toxicity of the core/shell-type nanorods against B16 and HSkMEC cells, and a negligible toxicity of the amine-modified core/shell-type nanomaterial against the same cells. Surface modification with –NH_2_ groups also made the final nanomaterial biocompatible and provided a possibility of binding/linking desired molecules to the surface of nanorods, via simple organic synthesis reactions. The non-cytotoxic nanomaterial modified with amine groups can be potentially used in many areas and techniques, e.g., biotechnology, bio-imaging, nanoengineering, drug delivery, fluorescent labeling, tracing techniques, etc.
